# Interference With the AMPKα/mTOR/NLRP3 Signaling and the IL-23/IL-17 Axis Effectively Protects Against the Dextran Sulfate Sodium Intoxication in Rats: A New Paradigm in Empagliflozin and Metformin Reprofiling for the Management of Ulcerative Colitis

**DOI:** 10.3389/fphar.2021.719984

**Published:** 2021-08-16

**Authors:** Mahmoud E. Youssef, Eslam E. Abd El-Fattah, Amir M. Abdelhamid, Hanan Eissa, Eman El-Ahwany, Noha A. Amin, Helal F. Hetta, Mohamed H. Mahmoud, Gaber El-Saber Batiha, Naglaa Gobba, Ahmed Gaafar Ahmed Gaafar, Sameh Saber

**Affiliations:** ^1^ Department of Pharmacology, Faculty of Pharmacy, Delta University for Science and Technology, Gamasa, Egypt; ^2^ Department of Biochemistry, Faculty of Pharmacy, Delta University for Science and Technology, Gamasa, Egypt; ^3^ Department of Clinical Pharmacology, Faculty of Medicine, Mansoura University, Mansoura, Egypt; ^4^ Department of Immunology, Theodor Bilharz Research Institute, Giza, Egypt; ^5^ Department of Hematology, Theodor Bilharz Research Institute, Giza, Egypt; ^6^ Department of Medical Microbiology and Immunology, Faculty of Medicine, Assiut University, Assiut, Egypt; ^7^ Department of Internal Medicine, College of Medicine, University of Cincinnati, Cincinnati, OH, United States; ^8^ Department of Biochemistry, College of Science, King Saud University, Riyadh, Saudi Arabia; ^9^ Department of Pharmacology and Therapeutics, Faculty of Veterinary Medicine, Damanhour University, Damanhour, Egypt; ^10^ Department of Pharmacology and Toxicology, College of Pharmacy, Misr University for Science and Technology, 6th of October City, Egypt; ^11^ Department of Pharmacology and Toxicology, Faculty of Pharmacy, Port-Said University, Port-Said, Egypt

**Keywords:** empagliflozin, metformin, ulcerative colitis, dextran sulfate sodium, AMPK, mTOR, NLRP3, IL-23/1L-17 axis 4

## Abstract

Empagliflozin and metformin are widely used for the treatment of type 2 diabetes. These drugs showed marked anti-inflammatory effects in different animal models *via* enhancing AMPK activity. Yet, the protective anti-inflammatory effects of their combination against ulcerative colitis have not been previously investigated. The current study aimed to explore the potential of empagliflozin/metformin combination to mitigate the DSS-induced rat colitis model. The modulating effects of empagliflozin and metformin on the AMPK/mTOR/NLRP3 axis and T cell polarization were delineated. In this study, distal colons were examined for macroscopic and microscopic pathological alterations. ELISA, qRT-PCR, and immunohistochemistry techniques were applied to detect proteins and cytokines involved in AMPK/mTOR/NLRP3 axis and T Cell polarization. Oral administration of empagliflozin (10 mg/kg/day) and metformin (200 mg/kg/day) combination alleviated colitis as revealed by the reduced disease activity index, macroscopic damage index, colon weight/length ratio, and histopathologic scoring values. Interestingly, empagliflozin/metformin combination significantly enhanced AMPK phosphorylation and depressed mTOR and NLRP3 expression leading to a subsequent reduction in caspase-1 cleavage and inhibition of several inflammatory cytokines, including IL-1β, and IL-18. Reduced mTOR expression and reduced IL-6 levels led to a reduction in Th17 cell polarization and maintenance. Together, the current study reveals that the protective effects of empagliflozin and metformin against DSS-induced colitis are fundamentally mediated *via* enhancing AMPK phosphorylation. Since adult humans with diabetes mellitus are at greater risk for developing inflammatory bowel diseases, clinical application of empagliflozin/metformin combination represents a novel therapeutic approach for treating diabetic patients with ulcerative colitis.

## Introduction

Ulcerative colitis is a chronic inflammatory bowel disease that is marked by increased intestinal motility and bloody diarrhea. The clinical value of existing therapeutic strategies of ulcerative colitis, including glucocorticoids, anti-tumor necrosis factor α (TNF-α), mesalamine, and thiopurines is still limited. Therefore, the discovery of new therapeutic approaches is essential to improve the effectiveness of the treatment (Ungaro et al., 2017; Saber et al., 2019). Adenosine monophosphate (AMP)-activated protein kinase (AMPK) is a conserved fuel-sensing enzyme that plays an important role in the regulation of cellular metabolism where it increases glucose and fatty acids uptake and activates the oxidation process to improve the cellular energy utilization (Marin-Aguilar et al., 2017). Additionally, AMPK activation is believed to counter many cellular disturbances such as inflammation, insulin resistance, and abnormal fat deposition (Ruderman et al., 2013). The diminished activity of AMPK and its failure to restore normal cellular energy levels is associated with these metabolic abnormalities and predisposed inflammation and apoptosis (Wu and Zou, 2020).

The mammalian target of rapamycin (mTOR) is a serine/threonine kinase that regulates cellular metabolism based on its need for differentiation and proliferation. The phosphorylated mTOR is formed from two characteristic proteins, mTOR complex 1 (mTORC1) and mTOR complex 2 (mTORC2) (Saxton and Sabatini, 2017). mTOR regulates the differentiation of T cells, as it accelerates the polarization of Th17 and Th1 cells and depresses regulatory T cell differentiation (Delgoffe et al., 2009). Additionally, the increased activation of mTOR leads to an elevation in inflammatory cytokines levels through differentiating type I macrophages (Li et al., 2020). Furthermore, the NLR family pyrin domain containing 3 (NLRP3) inflammasome is one of the main inflammatory pathways that is triggered by mTOR activation (Saber et al., 2020a; Saber and El-Kader, 2021).

Inflammasomes are multiprotein complexes that are directly involved in the inflammation process by the excessive activation of caspase-1, interleukin 1β (IL-1β), and interleukin 18 (IL-18) (Zheng et al., 2020; Saber et al., 2021a; Saber et al., 2021b; El-Rous et al., 2021). Five candidates of the pattern recognition receptor (PRR) participate in the inflammasome formation. These members are nucleotide-binding oligomerization domain (NOD), absent-in-melanoma 2 (AIM2), pyrin, leucine-rich repeat (LRR)-containing proteins (NLR) family (NLRP1, NLRP3) in addition to other reported PRRs including NLRP2, NLRP6, NLRP7, and NLRP12 (Kelley et al., 2019). Several inflammatory conditions are associated with NLRP3 inflammasome assembly and activation, including gout (Wang et al., 2019), hepatitis (Neumann et al., 2018), and ulcerative colitis (Saber et al., 2021a). It is believed that there is a crosstalk between AMPK, mTOR, and NLRP3 inflammasome signaling. Activation of AMPK reduces both mTOR and NLRP3 activity (Yang et al., 2019). Therefore, enhancing AMPK activity and its inhibitory role on mTOR/NLRP3 signaling could be an interesting therapeutic approach for combating inflammation.

Metformin is an antidiabetic biguanide drug that acts by promoting glucose uptake and utilization in the liver (Rena et al., 2017). Recent studies proved that metformin could possess other therapeutics benefits such as anti-aging (Soukas et al., 2019), anti-tumor (Aljofan and Riethmacher, 2019; Saber et al., 2020b), antifibrotic (Xiao et al., 2020), and anti-inflammatory effects (Saisho, 2015). By its ability to induce AMPK, metformin showed an anti-inflammatory effect in different inflammatory animal models. Metformin inhibited the inflammatory response in murine macrophages subjected to advanced glycation end products (AGE) by stimulating AMPK and suppressing nuclear factor kappa B (NF-κB)/receptor of advanced glycation end products (RAGE) pathway (Zhou et al., 2016). It also reduced colonic inflammation by AMPK-mediated inhibition of p38 mitogen-activated protein kinase (MAPK) and IL-6 (Di Fusco et al., 2018). The coloprotective effect of metformin was previously reported in several studies. Metformin alleviated the inflammatory response in the ulcerated colon by modulating sphingosine kinase 1/sphingosine 1 phosphate signaling pathway (El-Mahdy et al., 2021), reducing oxidative stress and downregulating transcription factor nuclear factor kappa B (NF-κB)-mediated pro-inflammatory signaling (Pandey et al., 2017) in addition to upregulating tissue growth factor β (TGF-β) (Liu et al., 2021). It also protected against intestinal barrier dysfunction by the inhibition of JNK activation *via* an AMPKα1-dependent signaling pathway (Deng et al., 2018). Moreover, metformin inhibited NLRP3 inflammasome activation *via* modulating AMPK-mTOR signaling in the heart of diabetic rats (Yang et al., 2019).

Empagliflozin is an antidiabetic drug that acts mainly by inhibiting sodium-glucose co-transporter-2 (SGLT-2) in the proximal tubules of the kidneys (Simes and MacGregor, 2019). It was revealed that SGLT-2 is widely expressed in rodents and human bodies. The immunohistochemical staining of different parts of the gastrointestinal tract proved that SGLT-2 is widely expressed along the gastrointestinal tract especially in small intestines and short epithelial portions of large intestines (Merigo et al., 2018). It was previously reported that empagliflozin could exhibit an anti-inflammatory effect through SGLT-2-independent mechanisms, including intracellular activation of AMPK, attenuation of superoxide production, and the suppression of oxidative stress (Zhou et al., 2018). Additionally, it showed an anti-inflammatory effect *via* the inhibition of NF-κB/JNK/STAT signaling pathways (Lee et al., 2021). Its suppressive effect on NFκB showed an anti-inflammatory cardioprotective effect in patients subjected to doxorubicin (Quagliariello et al., 1894). In diabetic patients, daily administration of empagliflozin ameliorated the inflammatory profile and promoted the antioxidant responses in leukocytes (Iannantuoni et al., 2019). However, the anti-inflammatory coloprotective role of empagliflozin is not fully elucidated and needs further investigations. In regard to the previous literature, our study aimed to examine the anti-inflammatory effect of AMPK activation by oral administration of empagliflozin or metformin in dextran sulfate sodium (DSS)-induced ulcerative colitis in rats.

## Results

### Effect on Colon Weight/Length Ratio, DAI, and MDI

**FIGURE 1:**
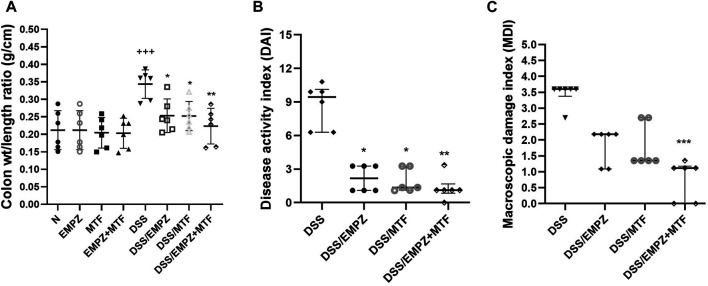
Effect on colon weight/length ratio, disease activity index (DAI), and macroscopic damage index (MDI) The effect of empagliflozin or metformin or their dual administration on **(A)** colonic weight/length ratio. **(B)** disease activity index. **(C)** macroscopic damage index. *n* = 6; 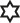
vs. DSS; ^Ⴕ^ vs. N; ns = non-significant. One symbol, two symbols, three symbols, and four symbols were assigned to indicate levels of significance at *p* < 0.05, *p* < 0.01, *p* < 0.001, and *p* < 0.0001, respectively.

The colon weight/length ratio was elevated in the DSS group compared with control groups or ulcerative groups treated with empagliflozin or metformin or their combination **(** Figure 1A). Treatment with empagliflozin or metformin or their combination significantly reduced the colonic weight to length ratio to reach the normal values. Similarly, the DAI was significantly elevated after DSS oral administration and was significantly declined after treatment with empagliflozin or metformin or their combination (Figure 1B). There was no significant difference in colonic weight to length ratio or DAI values between all treated ulcerative groups. The MDI value was significantly elevated in DSS, DSS/EMPZ, and DSS/MTF groups when compared with the ulcerative group that was treated with empagliflozin and metformin combination (Figure 1C).

### Histopathological Examination

**FIGURE 2:**
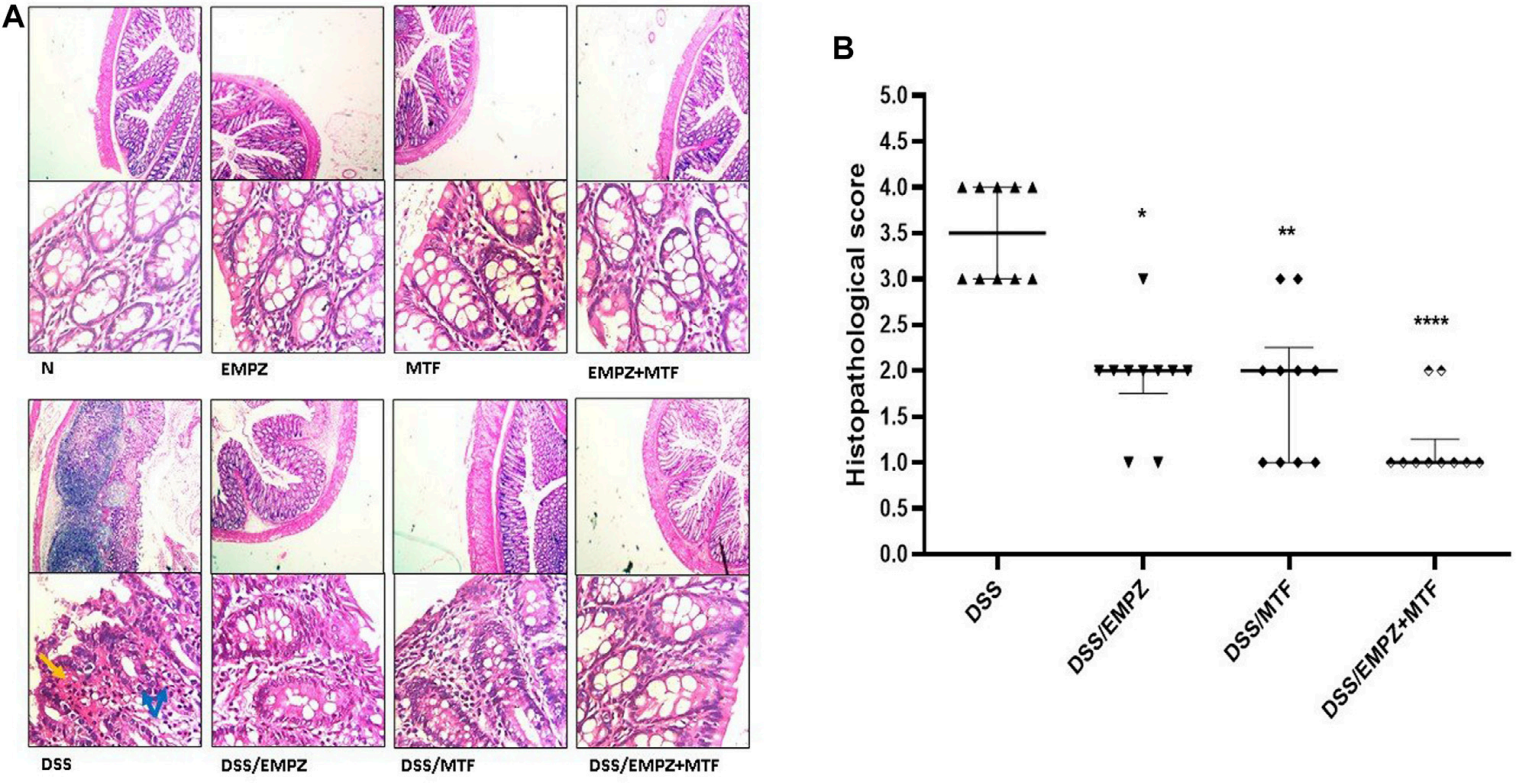
Histopathological examination **(A)** The effect of empagliflozin or metformin or their dual administration on histological scoring. **(B)** Colonic sections stained with H&E. DSS administration resulted in necrosis (black arrows), edema, and inflammatory cell infiltration (blue arrows). Treating DSS rats with empagliflozin or metformin or their combined therapy reestablished the normal colonic histology. 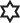
vs. DSS; One symbol, two symbols, and four symbols were assigned to indicate levels of significance at *p* < 0.05, *p* < 0.01, and *p* < 0.0001, respectively.

Oral DSS administration led to a distinct elevation in inflammatory cell infiltration and apparent disarray of the colonic tissue’s architecture, which resulted in the increased histopathological scoring in this group**.** Treatment with empagliflozin or metformin significantly reduced the histopathological scoring and a further decrease was obtained by the oral administration of their combination. However, there was no significant difference between DSS/EMPZ+MTF group when compared with other ulcerative groups that were treated with individual administration of empagliflozin or metformin (Figure 2A).

### Effect on OCLN and ZO-1

**FIGURE 3:**
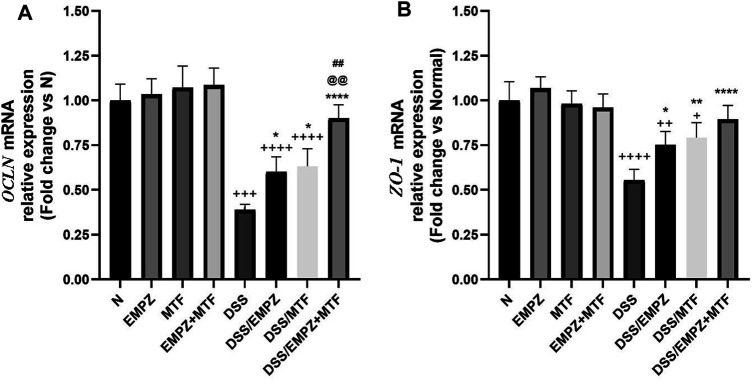
effect on occludin gene (OCLN) and zonula occludens-1 (ZO-1) The effect of empagliflozin or metformin or their dual administration on **(A)** *OCLN* mRNA levels. **(B)** *ZO-1* mRNA levels. *n* = 6; 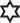
vs. DSS; ^Ⴕ^ vs. N; ^#^ vs. DSS/MTF; ^@^ vs. DSS/EMPZ. One symbol, two symbols, three symbols, and four symbols were assigned to indicate levels of significance at *p* < 0.05, *p* < 0.01, *p* < 0.001, and *p* < 0.0001, respectively.

The mRNA expression of OCLN was significantly decreased in the DSS group compared with healthy groups. Treatment with empagliflozin or metformin increased the expression of OCLN. The combination treatment with empagliflozin and metformin led to a significant elevation in OCLN levels when compared with DSS groups or other ulcerative groups that were treated with empagliflozin or metformin alone. Similarly, intoxication with DSS led to a marked reduction in ZO-1 mRNA expression, which was significantly elevated after empagliflozin or metformin individual administration without reaching normal levels (Figure 3B). The combined treatment with metformin and empagliflozin led to an increased ZO-1 expression to a level insignificantly different from DSS/EMPZ or DSS/MTF groups.

### Effect on SOD, ROS, GSH, MDA, and MPO Activity

**FIGURE 4:**
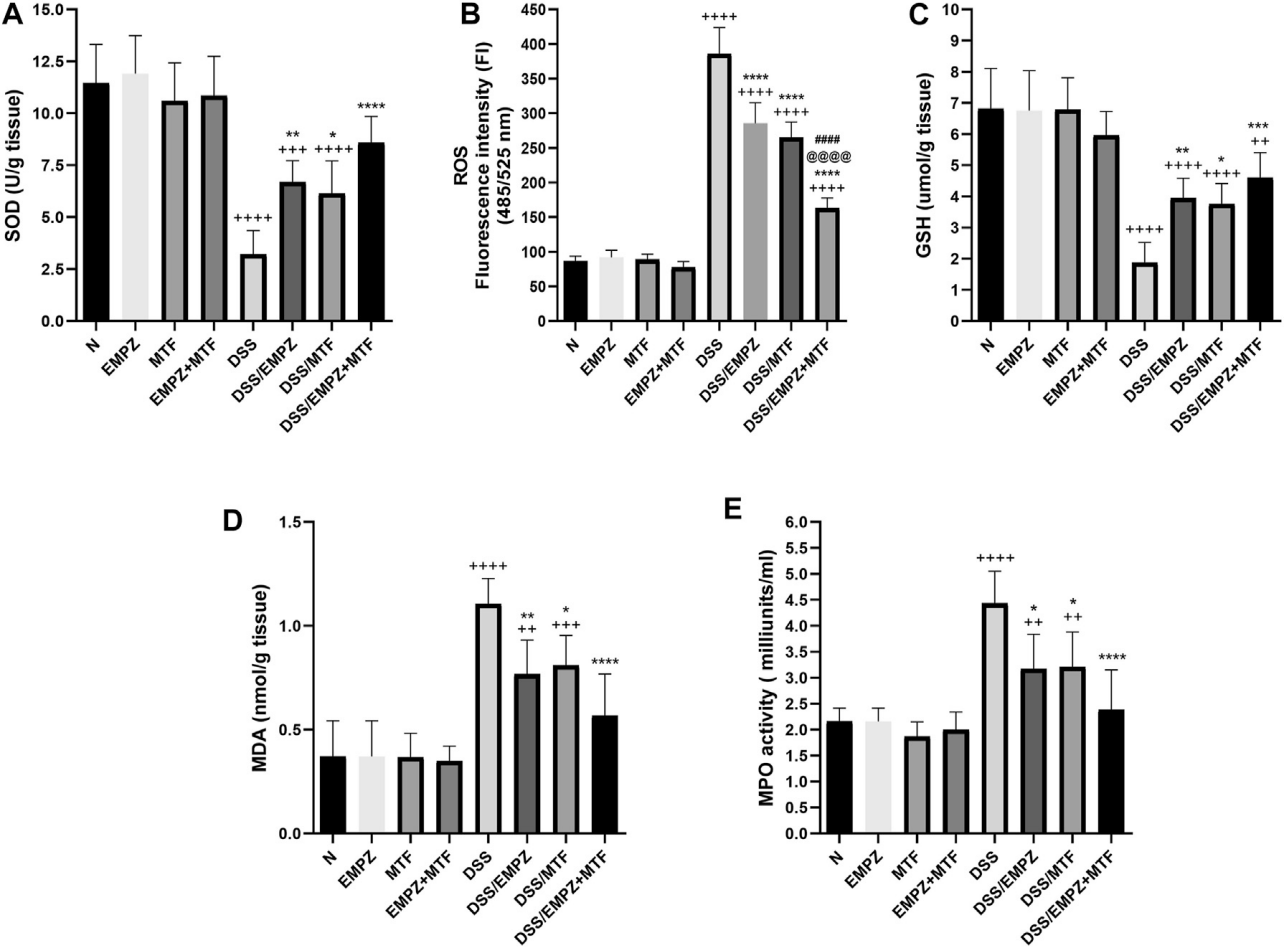
effect on superoxide dismutase (SOD), reactive oxygen species (ROS), glutathione (GSH), malondialdehyde (MDA), and MPO activity The effect of empagliflozin or metformin or their dual administration on **(A)** SOD levels. **(B)** ROS levels **(C)** GSH levels **(D)** MDA levels **(E)** MPO activity. *n* = 6; 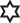
vs. DSS; ^Ⴕ^ vs. N; ^#^ vs. DSS/MTF; ^@^ vs. DSS/EMPZ. One symbol, two symbols, three symbols, and four symbols were assigned to indicate levels of significance at *p* < 0.05, *p* < 0.01, *p* < 0.001, and *p* < 0.0001, respectively.

The decreased antioxidant activity in untreated ulcerative rats was indicated by reduced SOD and GSH levels that were significantly restored by empagliflozin or metformin administration (Figures 4A,C). Normal levels of SOD, not GSH, were restored after treating ulcerative rats with empagliflozin/metformin combination. Additionally, daily administration of DSS increased the ROS levels in the colon compared to normal or treated ulcerative groups**.** Treating DSS rats with empagliflozin or metformin significantly increased the antioxidant activity in the colon and reduced ROS levels compared with the DSS rats. Administration of empagliflozin and metformin led to a further decrease in the colonic levels of ROS compared with DSS groups treated with individual administration of empagliflozin or metformin. However, the colonic levels of SOD in the DSS/EMPZ+MTF group are still above normal levels (Figure 4B). The results obtained from measuring MDA and MPO activity were parallel, where the daily administration of DSS led to a marked elevation in both markers (Figures 4D,E). Treating DSS rats with empagliflozin or metformin or their combination reduced MDA and MPO activity in the ulcerative colon without reaching normal levels. There was no significant difference in MDA and MPO activity between all treated ulcerative groups.

### Effect on p-AMPKα/AMPKα Ratio and p62

**FIGURE 5:**
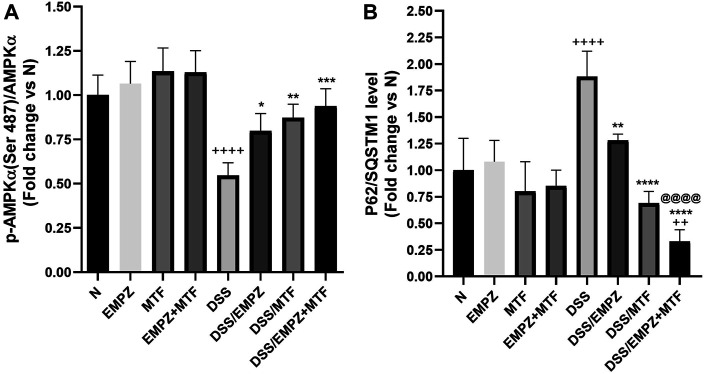
Effect on p-AMPKα/AMPKα and p62/SQSTM1 levels The effect of empagliflozin or metformin or their dual administration on **(A)** p-AMPKα levels. **(B)** p62/SQSTM1 levels. *n* = 6; 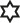
vs. DSS; ^Ⴕ^ vs. N; ^@^ vs. DSS/EMPZ. One symbol, two symbols, three symbols, and four symbols were assigned to indicate levels of significance at *p* < 0.05, *p* < 0.01, *p* < 0.001, and *p* < 0.0001, respectively.

As shown in Figure 5A, rats treated with DSS revealed a significant decline in the p-AMPK/AMPKα ratio compared to healthy control rats. The reduced levels of the p-AMPK/AMPKα ratio were significantly restored by treating DSS rats with empagliflozin or metformin or their combination compared with those of untreated ulcerative rats. There was no significant difference between all treated ulcerative groups. Oral administration of DSS increased the colonic tissue expression of p62 compared with normal groups (Figure 5B). Treating ulcerative rats with empagliflozin reduced the p62 levels to be comparable with normal values. Interestingly, combined administration of empagliflozin and metformin significantly reduced the expression of p62 compared with normal, DSS, and DSS/EMPZ groups.

### Effect on NLRP3 and Caspase-1

**FIGURE 6:**
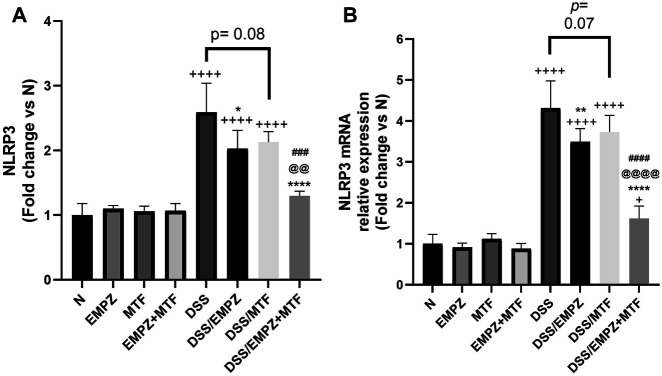
Effect on NLRP3 inflammasome The effect of empagliflozin or metformin or their dual administration on **(A)** NLRP3 levels in colonic tissue measured by ELISA (**B)** NLRP3 mRNA levels in colonic tissue measured by qRT-PCR. *n* = 6; 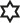
vs. DSS; ^Ⴕ^ vs. N; ^#^ vs. DSS/MTF; ^@^ vs. DSS/EMPZ. One symbol, two symbols, three symbols, and four symbols were assigned to indicate levels of significance at *p* < 0.05, *p* < 0.01, *p* < 0.001, and *p* < 0.0001, respectively.

**FIGURE 7:**
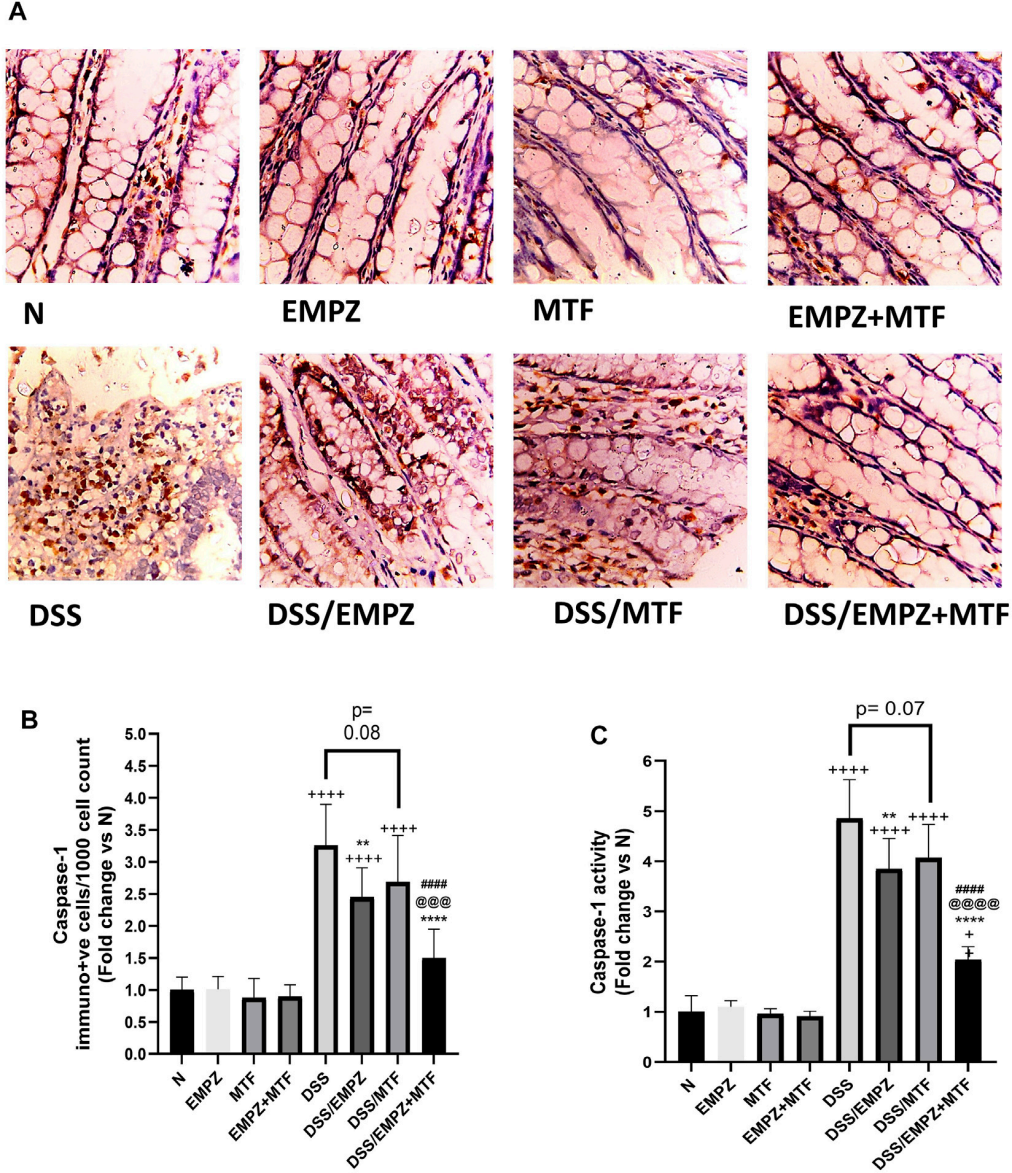
Effect on caspase-1 The effect of empagliflozin or metformin or their dual administration on **(A)** caspase-1 immunolabeling **(B)** caspase-1 immune-positive cells count **(C)** caspase-1 activity. *n* = 6; 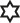
vs. DSS; ^Ⴕ^ vs. N; ^#^ vs. DSS/MTF; ^@^ vs. DSS/EMPZ. One symbol, two symbols, three symbols, and four symbols were assigned to indicate levels of significance at *p* < 0.05, *p* < 0.01, *p* < 0.001, and *p* < 0.0001, respectively.

Data obtained by applying ELISA or RT-PCR techniques to detect NLRP3 were parallel (Figures 6A,B)**.** NLRP3 levels were significantly elevated due to the irritating effect of DSS compared with normal groups. Daily treatment of ulcerative rats with empagliflozin significantly reduced NLRP3 protein and mRNA expressions. Surprisingly, individual administration of metformin did not significantly reduce NLRP3 tissue levels, and it was comparable to the level of the DSS rats. Combination treatment with empagliflozin and metformin significantly reduced both protein and mRNA expressions of NLRP3 (Figures 6A,B). Similarly, oral administration of DSS led to a marked elevation in caspase-1 expression and caspase-1 activity in untreated ulcerative rats (Figures 7A,B). The caspase-1 expression and caspase-1 activity were significantly reduced after treatment with empagliflozin, but their level was significantly different from normal levels. Individual treatment with metformin showed different findings where caspase-1 expression and caspase-1 activity were similar to the DSS group and significantly increased compared with normal groups. Empagliflozin/metformin combination significantly decreased caspase-1 expression and caspase-1 activity to reach normal levels. Comparison between the effect of individual administration of empagliflozin or metformin showed insignificant difference on NLRP3, caspase-1 expression, and caspase-1 activity (Figures 6A,B and Figures 7A,B).

### Effect on IL-1β and IL-18

**FIGURE 8:**
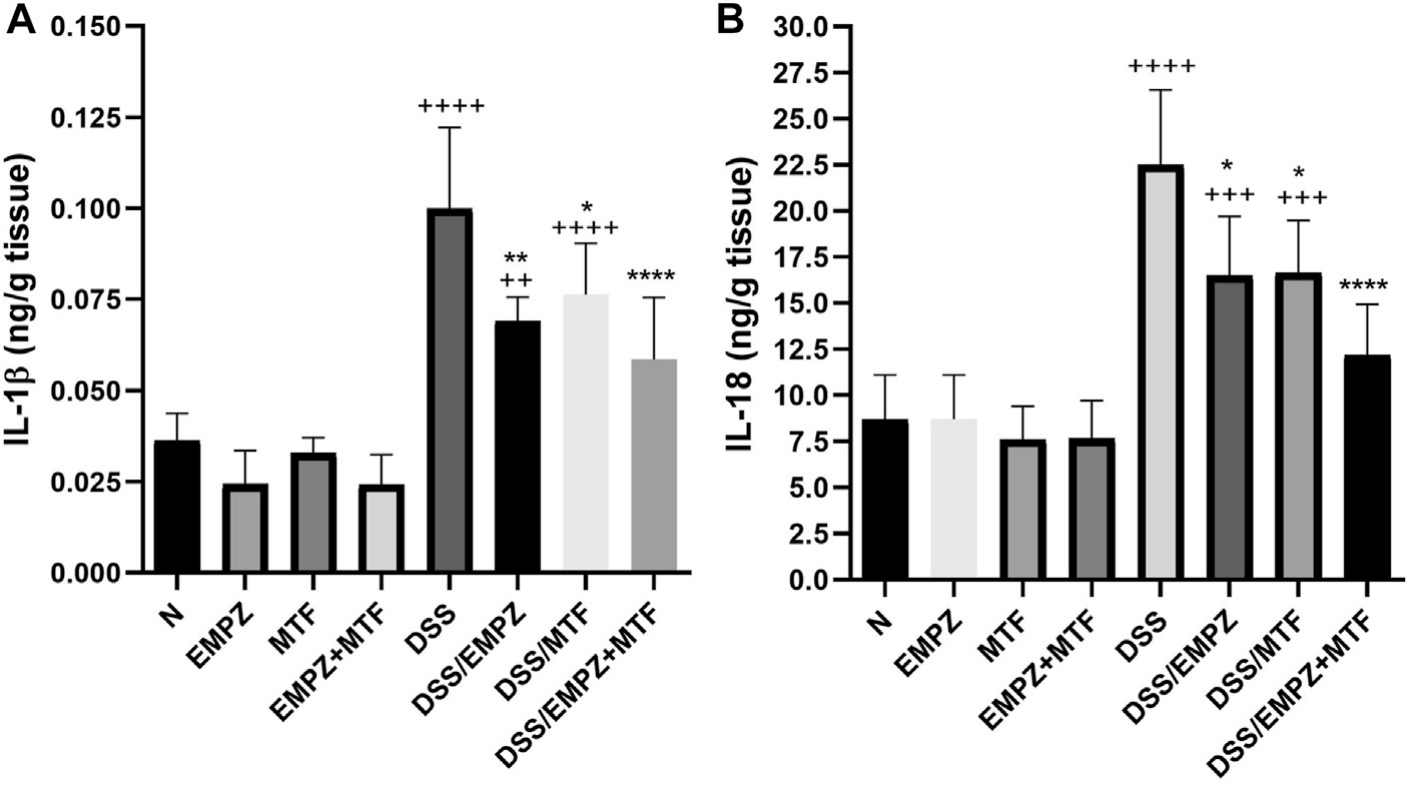
Effect on IL-1β and IL-18 The effect of empagliflozin or metformin or their dual administration on (**A)** IL-1β levels. **(B)** IL-18 levels. *n* = 6; 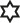
vs. DSS; ^Ⴕ^ vs. N; ^#^ vs. DSS/MTF; ^@^ vs. DSS/EMPZ. One symbol, two symbols, three symbols, and four symbols were assigned to indicate levels of significance at *p* < 0.05, *p* < 0.01, *p* < 0.001, and *p* < 0.0001, respectively.

The administration of DSS significantly elevated the colonic levels of IL-1β and IL-18 (Figures 8A,B). Treating DSS-intoxicated animals with empagliflozin or metformin revealed a significant decline in the levels of both cytokines. Dual administration of empagliflozin and metformin resulted in a reduction in IL-1β and IL-18 levels with insignificant difference compared to the DSS/EMPZ or DSS/MTF groups (Figures 8A,B). All treatment regimens that were given to ulcerative rats failed to reduce the tissue expression of IL-1β and IL-18 to normal levels.

### Effect on p-mTOR and Beclin-1

**FIGURE 9:**
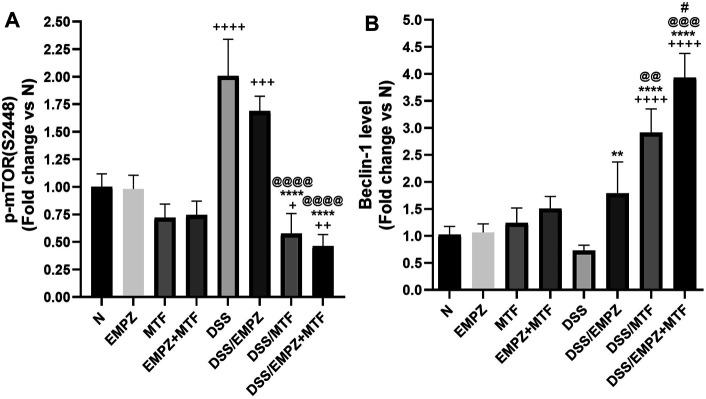
Effect on p-mTOR and beclin-1 The effect of empagliflozin or metformin or their dual administration on **(A)** p-mTOR levels **(B)** Beclin-1 levels. *n* = 6; 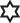
vs. DSS; ^Ⴕ^ vs. N; ^#^ vs. DSS/MTF; ^@^ vs. DSS/EMPZ. One symbol, two symbols, three symbols, and four symbols were assigned to indicate levels of significance at *p* < 0.05, *p* < 0.01, *p* < 0.001, and *p* < 0.0001, respectively.

The expression of p-mTOR was elevated in the colon of untreated ulcerative rats. Treating the DSS-intoxicated rat group with empagliflozin or metformin significantly reduced the p-mTOR levels. The daily administration of metformin, not empagliflozin, restored p-mTOR levels to comparable levels with that of the untreated normal control animals. Further, dual administration of empagliflozin and metformin to ulcerative rats led to a further decline in the p-mTOR level (Figure 9A). Although the tissue levels of beclin-1 were reduced in the untreated DSS rat group, there was an insignificant difference compared to the healthy untreated group. Treatment of ulcerative rats with empagliflozin significantly elevated beclin-1 levels when compared with DSS group. Administration of metformin or empagliflozin/metformin combination led to a further increase in the tissue levels of beclin-1 to be significantly different from the normal and DSS/EMPZ groups (Figure 9B).

### Effect on IL-17A, IL-23, IL-6, and IL-10

**FIGURE 10:**
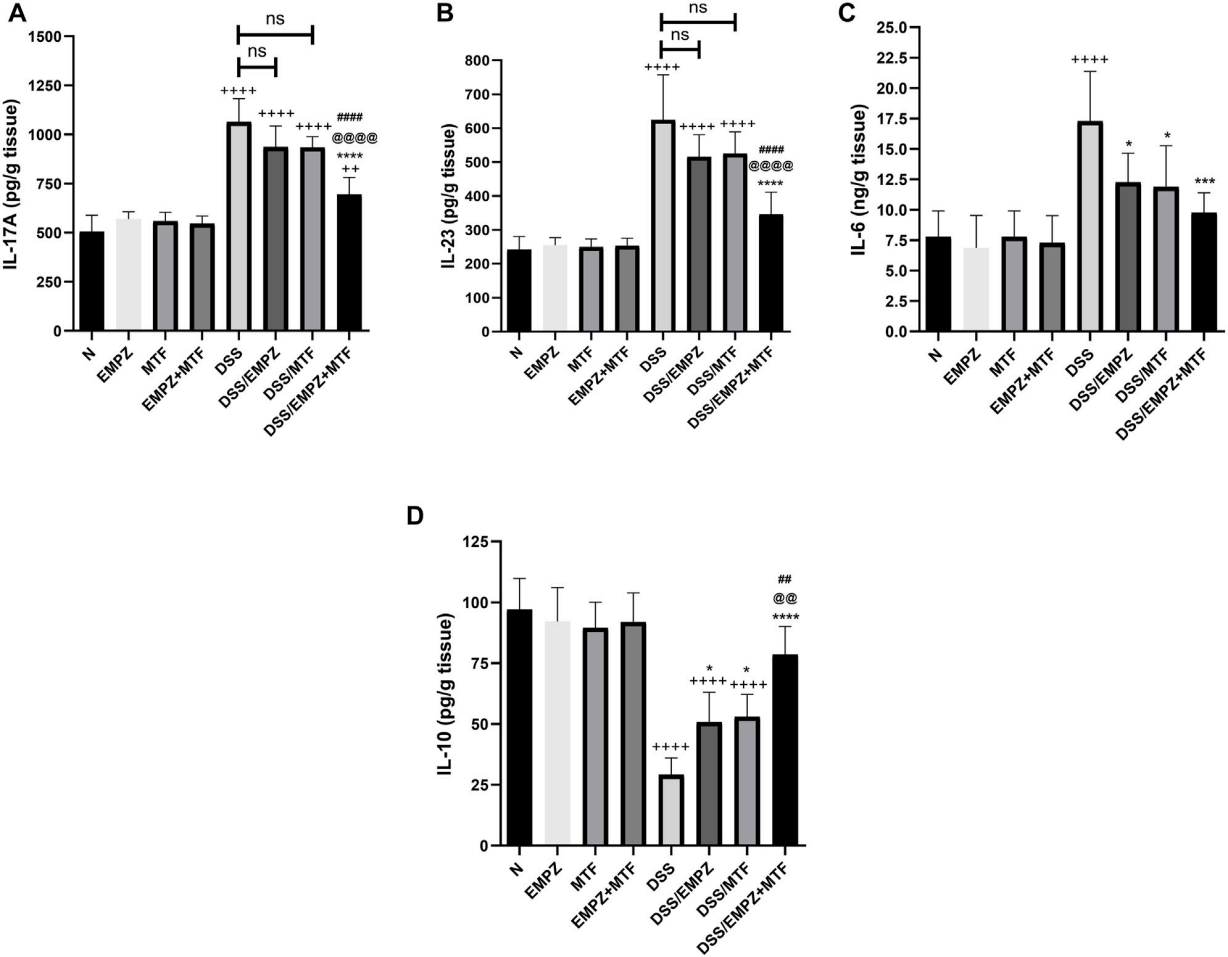
Effect on IL-17A, IL-23, IL-6, and IL-10 The effect of empagliflozin or metformin or their dual administration on **(A)** IL-17A (**B)** IL-23 **(C)** IL-6 days. IL-10. *n* = 6; 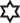
vs. DSS; ^Ⴕ^ vs. N; ^#^ vs. DSS/MTF; ^@^ vs. DSS/EMPZ. One symbol, two symbols, three symbols, and four symbols were assigned to indicate levels of significance at *p* < 0.05, *p* < 0.01, *p* < 0.001, and *p* < 0.0001, respectively.

The levels of IL-17A (Figure 10A) and IL-23 (Figure 10B) were increased as a result of the DSS intoxication. Treating DSS rats with empagliflozin or metformin reduced the levels of IL-17A and IL-23 to a one that was insignificantly different compared to the untreated ulcerative group. Combination treatment with empagliflozin and metformin revealed a significant decrease in the levels of IL-17A and IL-23 compared with the untreated ulcerative group or ulcerative groups treated with individual administration of empagliflozin or metformin. The empagliflozin/metformin combination restored normal IL-23, not IL-17A, tissue levels (Figures 10A,B). Similarly, DSS-induced irritation to the colon increased to the formation of IL-6. However, administration of empagliflozin or metformin or their combination reduced the colonic expression of IL-6 without reaching the normal levels. There was no significant difference in IL-6 levels between different treated ulcerative groups (Figure 10C). DSS oral administration significantly decreased the expression of IL-10, the anti-inflammatory cytokine. The level of IL-10 was elevated in ulcerative groups that were treated with either empagliflozin or metformin (Figure 10D). However, the oral administration of empagliflozin/metformin combination restored normal levels of IL-10 and their effect on IL-10 colonic levels was significantly different from DSS/EMPZ or DSS/MTF groups (Figure 10D).

## Discussion

Ulcerative colitis is a recurrent inflammatory bowel disease that affects the large intestine and has an increasing incidence rate worldwide (Rubin et al., 2019). The current therapeutic strategies are effective at reducing the disease progression and relieving the symptoms of the disease (Torres et al., 2013). In the present research, we investigated the effect of metformin, empagliflozin, or their combined therapy in a DSS-induced murine model of ulcerative colitis. We performed microscopic and macroscopic examinations of the colon to evaluate the colon weight/length ratio, MDI, and DAI. We investigated AMPK, mTOR, and NLRP3 colonic levels in addition to the elucidation of caspase-1 activity and several inflammatory cytokines, including IL-1β, IL-18, IL-23, and IL-17.

DSS-induced model of ulcerative colitis is widely applied as it morphologically and symptomatically resembles that what happens in humans (Eichele and Kharbanda, 2017). In this model, DSS acts by direct chemical intoxication of the epithelial layer of the colon resulting in its disruption allowing invasion by luminal bacterial antigens into the intestinal mucosa (Eichele and Kharbanda, 2017) leading to severe histopathological and morphological changes, which were reported in our study and represented by the high DAI, MDI, and histopathological scoring in the untreated ulcerative group.

OCLN and ZO-1 are plasma membrane proteins that participate in the formation and stabilization of tight junctions between epithelial cells (Diaz-Coranguez et al., 2019). The DSS-induced intoxication of the colon will result in a marked depression in both proteins, which would increase the permeability of intestinal contents and the subsequent progression of inflammatory response with typical features of ulcerative colitis such as tissue necrosis, severe infiltration of inflammatory cells, bleeding, and edema (Landy et al., 2016). However, in the present research, the determination of OCLN and ZO-1 by their mRNA levels has a limited value, because these proteins have important post-transductional regulation.

The toxic effects of DSS could reduce the activity of glutathione reductase and glutathione peroxidase in addition to its role in inducing lipid peroxidation and oxidative stress (Chen et al., 2019). This could explain the reduced GSH and SOD in the current ulcerative colitis model. Additionally, the reported cellular damage of the colon led to an elevation in MDA levels, which serves as an indicator of tissue decomposition in response to lipid peroxidation and an indicator of oxidative cellular damage (Ayala et al., 2014).

The decreased phosphorylation of AMPK in the current model could be attributed to the cellular stress in inflammatory conditions (Gauthier et al., 2011). One of the major downstream pathways that are regulated by AMPK is the mTOR/NLRP3 inflammasome pathway. The reduced phosphorylation of AMPK could lead to an elevation in mTOR, which was previously reported in several studies (Zhao et al., 2018; Qing et al., 2019; Yang et al., 2019). AMPK phosphorylates at least two sites in mTORC1, the mTOR binding partner raptor and the tuberous sclerosis protein 2 (TSC2) tumor suppressor leading to its suppression. Due to the decreased activity of AMPK in the current model of ulcerative colitis, the mTOR activity was enhanced, which also led to an activation in NLRP3 inflammasome expression. This could be attributed to the reported role of mTOR in regulating the activation and assembly of NLRP3 inflammasome *via* ROS-induced NLRP3 expression (Li et al., 2018). Another mechanism of mTOR-mediated activation of NLRP3 inflammasome could be due to the induction of glycolysis, which represents an important metabolic pathway in the activation of NLRP3 inflammasome (Moon et al., 2015). The reported increase in caspase-1 activity and the subsequent elevation in IL-1β and IL-18 in the current model is due to the increased NLRP3 inflammasome activity.

Beclin-1, the mammalian ortholog of yeast Atg6, is usually elevated in response to cellular stress and depressed during the cell cycle. The reduction of beclin-1 expression would enhance the production of IL-1β and IL-18 due to impaired autophagic response (Houtman et al., 2019), which was observed in our study. Furthermore, AMPK regulates beclin-1 phosphorylation and stimulates autophagy (Zhang et al., 2016). Therefore, the reduced beclin-1 expression in the current model of ulcerative colitis could be due to the diminished AMPK activity. p62 is an autophagosome cargo protein encoded by the SQSTM1 gene. It selectively targets and binds to other proteins for autophagy, including several inflammatory proteins such as TNF receptor-associated factor 6 (TRAF6), Nrf2, and inflammasome (Tilija Pun and Park, 2017). In our study, p62 was elevated due to DSS-induced inflammatory response; however, its mediated-autophagic activity failed to reduce the NLRP3 inflammasome tissue levels in DSS-intoxicated rats treated with metformin. Therefore, future studies are needed to explain the crosstalk between mTOR, P62, and NLRP3 signaling in different inflammatory and ischemic conditions.

The differentiation of Th17 cells is regulated by multiple signaling mechanisms and a complex network of transcription factors (Jiang et al., 2019). Recently, it was proven that mTOR complexes (mTORC1 and mTORC2) are directly involved in the differentiation of CD4 T-cells into Th17 cells and their differentiation is diminished in the absence of mTOR (Waickman and Powell, 2012). Additionally, the generation and polarization of Th17 cells are mediated by IL-6 and transforming growth factor β (TGF-β) where they participate in the expression of the master transcription factor RORγt. The maintenance and expansion of Th17 cells are controlled by a heterodimeric cytokine known as IL-23 that stabilizes Th17 cells by binding to their master transcription factor RORγt and facilitate their function in releasing effector cytokines such as IL-17 and IL-21 (Martinez et al., 2008; Rafa et al., 2013; Cătană et al., 2015; Schmitt et al., 2021). In the current ulcerative colitis model, the increased expression of mTOR and IL-6 in the colon of ulcerative rats enhanced the generation of Th17 cells and the release of IL-17 and IL-23 in line with reducing the expression of IL-10, the anti-inflammatory cytokine.

Recently, it was reported that metformin and empagliflozin have an anti-inflammatory effect *via* promoting the phosphorylation and activation of AMPK (Lu et al., 2019). Moreover, the hypoglycemic effect of metformin is mainly mediated by reducing the production of ATP by mitochondria and increasing the AMP/ATP in the cytoplasm, which leads to metabolic changes that stimulate AMPK (Rena et al., 2017). Daily oral administration of metformin resulted in an activation of AMPK that was accompanied by an obvious decrease in mTOR expression. Despite the reported stimulatory effect of empagliflozin on AMPK phosphorylation, its administration failed to suppress the mTOR activity. The superiority of metformin over empagliflozin in suppressing mTOR activity could be due to its role in inhibiting mTOR *via* AMPK-independent mechanisms such as Rag GTPases-dependent suppression of mTORC1 signaling (Kalender et al., 2010). Concerning their effect on NLRP3 inflammasome, it was previously reported that metformin had a significant inhibitory effect on NLRP3 (Bullón et al., 2016; Yang et al., 2019; Guo et al., 2020), which is opposite to our findings; in which metformin had an insignificant effect on the NLRP3 on the protein and the gene expression levels. In our study, individual administration of both drugs showed minimal inhibitory effect on NLRP3 inflammasome and the subsequent inhibition of caspase-1 activation, IL-1β, and IL18. This seems to be dependent on the experimental design, the administered dosage and the duration of therapy. However, the combined administration of both drugs bypassed the decreased efficacy of their individual administration. Empagliflozin/metformin combination strongly activated AMPK phosphorylation and suppressed mTOR expression leading to a robust inhibitory effect on NLRP3 inflammasome assembly and caspase-1 cleavage.

Individual administration of empagliflozin did not significantly reduce IL-6 expression and mTOR expression, which was reflected in the reported increase in the production of IL-17 from Th17 cells. Although individual administration of metformin significantly decreased the levels of mTOR, it showed a minimal inhibitory effect on IL-6. As a result of these two actions, IL-17 production was elevated. The increased polarization of Th17 cells after individual administration of empagliflozin or metformin also led to an increase in the colonic levels of IL-23 and a suppression in IL-10, the anti-inflammatory cytokine (Bunte and Beikler, 2019). On the contrary, combined administration of metformin and empagliflozin showed a marked suppressor effect on Th17 cell polarization and stabilization. Empagliflozin/metformin combination significantly decreased the levels of IL-6 and mTOR, an action that lead to a consequent suppression in IL-17 and IL-23 production, indicating a minimized polarization and stabilization of Th17 cells.

## Conclusion

The combined administration of empagliflozin and metformin demonstrated a coloprotective anti-inflammatory effect in the DSS-intoxicated rats. Daily oral administration of empagliflozin/metformin combination has improved the macroscopic and microscopic characteristics of the ulcerated colon due to stimulation of AMPK phosphorylation. The empagliflozin/metformin combined therapy exhibited greater protective and ameliorative effects, compared to their monotherapies, on ulcerative colitis by inducing AMPK-induced suppression of mTOR/NLRP3 inflammasome axis resulting in the inhibition of downstream activation of caspase-1 and other inflammatory cytokines. Additionally, their role in suppressing IL-6 and mTOR reduced the differentiation and polarization of Th17 cells. Such a novel therapeutic approach offers a basis for future application for treating ulcerative colitis in humans. It might also be encouraging in the treatment of other inflammatory conditions, particularly in diabetic patients.

## Materials and Methods

### Animals

Adult male Wister rats were purchased from the animal facility at the Delta University for Science and Technology, Egypt. Rats (weighing 200–250 g) were housed and maintained under standard environmental conditions with ad lib access to water and food. Protocols were approved by the corresponding Institutional Animal Care and Use Committee at the Faculty of Pharmacy, Delta University for Science and Technology (approval no, FPDU12520/3).

### Experimental Design

In the first 2 days, empagliflozin (10 mg/kg/day p.o.), metformin (200 mg/kg/day p.o.), or the empagliflozin/metformin combination at the same doses were given to three different groups before the induction of ulcerative colitis. Additional three normal healthy groups were designed to represent the control groups and administered an identical regimen**.** On the third day, the assigned ulcerative groups were subjected to daily oral doses of (DSS) 4% w/v for 1 week in addition to resuming the oral administration of the treatment regimen for each group. The oral daily administration of empagliflozin or metformin or their combined therapy were continued for 1 week after the discontinuation of DSS administration (Table 1). Based on previous reports, doses of metformin (Simanenkova et al., 2021) and empagliflozin (Shao et al., 2019) were selected.

**TABLE 1:** Experimental design.

Exp. groups	n	Day 1–day 2	Day 3–day 9	Day 10–day 16
N	6	—	—	—
EMPZ	6	Empagliflozin (10 mg/kg/day, p.o.)	Empagliflozin (10 mg/kg/day, p.o.)	Empagliflozin (10 mg/kg/day, p.o.)
MTF	6	Metformin (200 mg/kg/day, p.o.)	Metformin (200 mg/kg/day, p.o.)	Metformin (200 mg/kg/day, p.o.)
EMPZ+MTF	6	Empagliflozin (10 mg/kg/day, p.o.) + Metformin (200 mg/kg/day, p.o.)	Empagliflozin (10 mg/kg/day, p.o.) + Metformin (200 mg/kg/day, p.o.)	Empagliflozin (10 mg/kg/day, p.o.) + Metformin (200 mg/kg/day, p.o.)
DSS	8	—	4% DSS in drinking water	—
DSS/EMPZ	6	Empagliflozin (10 mg/kg/day, p.o.)	Empagliflozin (10 mg/kg/day, p.o.) + 4% DSS in drinking water	Empagliflozin (10 mg/kg/day, p.o.)
DSS/MTF	6	Metformin (200 mg/kg/day, p.o.)	Metformin (200 mg/kg/day, p.o.) + 4% DSS in drinking water	Metformin (200 mg/kg/day, p.o.)
DSS/EMPZ+MTF	6	Empagliflozin (10 mg/kg/day, p.o.) + Metformin (200 mg/kg/day, p.o.)	Empagliflozin (10 mg/kg/day, p.o.) + Metformin (200 mg/kg/day, p.o.) + 4% DSS in drinking water	Empagliflozin (10 mg/kg/day, p.o.) + Metformin (200 mg/kg/day, p.o.)

### Chemical and Materials

Empagliflozin, metformin, and DSS were purchased from Sigma-Aldrich (St. Louis, MO, United States). Detection of the p-AMPKα/AMPKα ratio was performed using an ELISA kit obtained from RayBiotech (Norcross, GA, United States). ubiquitin-binding protein 62 (P62/SQSTMI “p62”) colonic levels were assessed using an ELISA kit purchased from MyBioSource (CA, United States). ELISA detection kits obtained from Cell Signaling Technology (MA, United States) and CUSABIO (Wuhan, China) were used for the determination of mTOR and Beclin-1, respectively. For the detection of interleukin 6 (IL-6), and interleukin 10 (IL-10) cytokines, ELISA kits were obtained from eBioscience (Vienna, Austria) while IL-17A and IL-23 were detected by ELISA using kits purchased from CUSABIO. NLRP3 was measured by MyBioSource ELISA detection kit. Myeloperoxidase enzyme (MPO), malondialdehyde (MDA), glutathione (GSH), and superoxide dismutase (SOD) were detected spectrophotometrically using kits obtained from Biodiagnostic (Cairo, Egypt). kits purchased from R&D Systems (Minneapolis, MN, United States) were used for the detection of caspase-1 activity and reactive oxygen species (ROS). The primary antibody that was used in the immunohistochemical staining of caspase-1 was purchased from CUSABIO. The EnVision™ FLEX immunohistochemistry detection kit was obtained from Dako Inc. (Glostrup, Denmark).

### Assessment of the Colon Weight/Length Ratio

Animals’ colons were separated from the caecum to the anus, flushed with phosphate-buffered saline (PBS), dried by clean towels, and emptied before their weighting. The colon length was measured, and the colon weight/length ratio was calculated for each rat.

### Collection of Samples

At the end, rats were anesthetized, sera were collected and preserved at −80°C. After that, animals were sacrificed, colons were separated, cleaned, dissected into two portions: one was preserved in natural-buffered formalin (4%) and fixed in paraffin after 24 h for histological and IHC investigations. The second was immediately dipped into liquid nitrogen and stored at −80°C for subsequent ELISA, RT-PCR, and spectrophotometric assays of different markers. The colonic tissue homogenate was prepared by placing 10% w/v colonic tissue in 20 mM of Tris-HCl and 1 mM EDTA solution. After sonication, the homogenate was centrifuged at 4 °C at a speed of 3,000 revolutions per minute. The supernatant was kept immediately at −80°C for further investigations.

### Assessment of Disease Activity Index

A scoring system (0–4) was applied for the estimation of the irritating effect of DSS accounting on alterations in weight, stool consistency, and the presence of blood. Animals that showed no percentage decrease in body weight were given a score (0). Scores (1), (2), (3), and (4) were given if the animals showed a decrease in body weight by 1, 6, 11, and 21%, respectively. Regarding the stool consistency and the presence of bloody stool, the following score system was applied: score (0), normal stool and the absence of bloody stool; score (1–2), presence of loose stool with mild bleeding; score (3–4), appearance of watery diarrhea with excessive bleeding.

### Calculation of the Macroscopic Damage Index

A scoring system (0–4) was applied to assess the macroscopic damage. MDI was elucidated in a single-blind technique. MDI scoring based on the observation of multiple macroscopic profiles as follows: score (0), absence of pathological macroscopic features; score (1), mucosal erythema only; score (2), mild mucosal bleeding, and slight mucosal edema; score (3), moderate mucosal bleeding and edema; score (4), severe mucosal bleeding and edema.

### Histopathological Examination

The paraffin blocks of the colonic tissue were cut into 4 μm-thick sections and stained with hematoxylin and eosin (H&E) for histopathological evaluation. A blinded histopathologist applied a scoring system (0–4) to evaluate the histopathological abnormalities or lesions along the entire colon as the following: score (0), absence of any histopathological features; score (1), presence of mild inflammation; score (2), mild infiltration of inflammatory cells; score (3), moderate infiltration of inflammatory cells, which is associated with elevated vascularity and increased colonic thickness; score (4), severe loss of goblet cells, severe infiltration of inflammatory cells with high vascularity and increased colon thickness.

### Immunohistochemical Analysis of Caspase-1

Following the Tris/EDTA buffer (pH = 8) antigen retrieval protocol, the caspase-1 primary polyclonal antibody was diluted by PBS (1:100). The diluted caspase-1 primary antibody was added to the deparaffinized sections of the colon and incubated at 4°C overnight. A dextran-secondary antibody was added and formed coupled molecules were stained using DAB chromogen substrate. Mayer’s hematoxylin was added to concentrate the resulting color. Positive immune reactions (brown) for caspase-1 were recorded (40X). Finally, the positive immune reactions of caspase-1 were detected by calculating the immunopositive cells per 1,000 cell count, and the values are expressed as fold change vs that of the N group.

### Determination of p62, Beclin-1, NLRP3, and Inflammatory Cytokines

Tissue levels of phosphorylated p62/SQSTM1, NLRP3, beclin-1, IL-1β, IL-18, IL-17, IL-23, IL-6, and IL-10 were measured by applying quantitative sandwich ELISA technique according to the manufacturer’s protocol.

### Determination of p-AMPKα/AMPKα

As instructed, a homogenization step was performed using Lysis Buffer #9803 for lysis of colon tissue samples at a ratio of 100 mg of distal colon tissue to 1 ml of buffer. Followed by centrifugation of the tissue lysate. The buffer contained 20 mM Tris-HCl (pH 7.5), 150 mM NaCl, 1 mM Na2EDTA, 1 mM EGTA, 1% Triton, 2.5 mM sodium pyrophosphate, 1 mM beta-glycerophosphate, 1 mM Na3VO4, and 1 μg/ml leupeptin in the presence of protease inhibitor cocktail and phosphatase inhibitor in addition to 1 mM PMSF immediately before use. The sample protein concentration in the extract was determined using BCA protein assay reagent kit purchased from Thermo Fisher Scientific Inc (Rockford, United States) and samples were diluted to the desired concentration using the buffer. An anti-pan AMPKα antibody has been coated onto a 96-well plate. Samples were pipetted into the wells and AMPKα present in a sample is bound to the wells by the immobilized antibody and the wells were washed. In select wells, rabbit antiphospho-AMPKα (Ser 487) antibody was added to detect phosphorylated AMPKα. In the remaining wells, biotinylated anti-pan-AMPKα antibody was used to detect pan AMPKα. After washing away unbound antibody, HRP-conjugated anti-rabbit IgG or HRP-Streptavidin was pipetted into the wells. The wells were again washed, a TMB substrate solution is added to the wells and color develops in proportion to the amount of AMPKα (Ser 487) or pan AMPKα bound. Data were expressed as normalized p-AMPKα/AMPKα.

### Determination of p-mTOR (S2448)

Tissue lysates were prepared as abovementioned, the sample protein concentration in the extract was determined using BCA protein assay reagent kit and the samples were diluted to the desired concentration using the buffer. a solid phase sandwich ELISA that detects endogenous levels of mTOR protein phosphorylated at S2448. An mTOR mouse antibody has been coated onto the microwells. After incubation with lysates, mTOR (phospho and nonphospho) protein is captured by the coated antibody. Following extensive washing, a phospho-mTOR (S2448) rabbit antibody was added to detect the captured phospho-mTOR protein. Anti-rabbit IgG, HRP-linked antibody was then used to recognize the bound detection antibody. HRP substrate, TMB is added to develop color. The magnitude of the absorbance for this developed color is proportional to the quantity of mTOR phosphorylated at S2448. Data were expressed as normalized optical density values. In general, the ELISAs could surpass the Western Blot (WB) as the values of standard error are much smaller than those of the WB. WB is to some degree used to quantify but is not nearly as reliable as ELISA for that purpose. Additionally, ELISA does not go through the process of electrophoresis which may encounter problems during the preparation of the gel. Further, we performed a protein quantification step which implies the accuracy of the assays.

### Determination of MPO, GSH, SOD, MDA, ROS, and Caspase-1 Activity

MPO catalyzes the production of hypochlorous acid, which reacts with taurine to form taurine chloramine. TNB reacts with taurine chloramine to form a colorless product (DTNB). Therefore, the decrease in color intensity is an indication of higher MPO activity and concentration. Regarding the detection of caspase-1 activity, we applied a colorimetric technique that is based on the ability of caspase-1 to cleave p-nitroanilide to yellow product and detected at 405 nm. For the determination of MDA, incubation of tissue homogenate and thiobarbituric acid for 30 min at 95°C in acidic media will result in a thiobarbituric reactive product with pink color that is measured spectrophotometrically at 534 nm. GSH, and SOD were determined according to the manufacturer’s instructions. For the determination of ROS, briefly, distal colon tissue (200 mg) was homogenized in ice-cold Tris-HCl buffer (40 mM, pH = 7.4) (1:10 w/v). Then, homogenates (100 µl) were mixed with Tris-HCl buffer (1 ml), and 5 µl of 20, 70-dichlorofluorescein diacetate (10 µM) (Sigma-Aldrich) was added and the reactions were incubated for 30 min at 37°C. The fluorescence intensity (FI) was measured with a SpectraFluor Plus Microplate Reader Tecan, Mainz, Germany), λ excitation = 485 nm and λ emission = 525 nm.

### qRT-PCR Analysis for the Expression of *OCLN*, Zonula Occludens-1 , and NLRP3 mRNA

For the extraction of mRNA from the colonic sections, an RNeasy mini kit, obtained from Qiagen (Netherlands, Germany) was used. The purity and concentration of mRNA were detected by a nanodrop at 260 nm. Complementary DNA was produced by reversing mRNA using a specified kit that was purchased from Qiagen. qRT-PCR was performed using a thermocycler with SYBR Green PCR Master Mix (Qiagen). The comparative cycle threshold (Ct) (2^−ΔΔCT^) was applied to calculate the relative expression of each gene. The nucleotides’ sequences for each primer pairs are summarized in Table 2.

**TABLE 2:** Primer sequences for qPCR.

Primer	GenBank accession	F	R	Amplicon size (bp)
*OCLN*	NM_031329.3	5′-CTG​TCT​ATG​CTC​GTC​ATC​G-3′	5′-CAT​TCC​CGA​TCT​AAT​GAC​GC-3′	294
*ZO-1*	XM_017588936.1	5′-GCC​TCT​GCA​GTT​AAG​CAT-3′	5′-AAG​AGC​TGG​CTG​TTT​TAA-3′	249
NLRP3	NM_001191642.1	5′-GAG​CTG​GAC​CTC​AGT​GAC​AAT​GC-3′	5′-ACC​AAT​GCG​AGA​TCC​TGA​CAA​CAC-3′	146
GAPDH	NM_017008.4	5′-TCA​AGA​AGG​TGG​TGA​AGC​AG-3′	5′-AGG​TGG​AAG​AAT​GGG​AGT​TG-3′	111

### Statistical Analysis

GraphPad Prism software version 8 (GraphPad Software Inc., La Jolla, CA, United States) was used to statistically analyze data. Comparisons between different groups were performed using one-way ANOVA followed by Tukey’s posthoc test. For the multiple comparisons of differences in scoring systems among groups, the Kruskal-Wallis test and Dunn’s posthoc test were applied. Data are expressed as the mean ± (SD). A value of probability (P) < 0.05 was statistically significant. Using G*power 3.1.9.7 and given α, sample size, and effect size, we performed a post-hoc power analysis and found that the calculated power (1-β) = 0.9.

## Data Availability

The original contributions presented in the study are included in the article/supplementary material, further inquiries can be directed to the corresponding author.

## Abbreviations

AIM2, absent-in-melanoma 2; AMP, adenosine monophosphate; AMPK, adenosine monophosphate (AMP)-activated protein kinase; DAB, 3,3′-diaminobenzidine; DAI, Disease activity index; DSS, dextran sulfate sodium; GSH, glutathione; IL-6, interleukin 6; LRR, leucine-rich repeat; MDA, malondialdehyde; MDI, macroscopic damage index; MPO, myeloperoxidase enzyme; mTOR, mammalian target of rapamycin; mTORC1, mTOR complex 1; mTORC2, mTOR complex 2; NF-κB, nuclear transcription factor kappa B; NLR, leucine-rich repeat-containing proteins; NLRP3, NOD like receptor family pyrin domain containing 3; NOD, nucleotide-binding oligomerization domain; p38 MAPK, p38 mitogen-activated protein kinase; RAGE, receptor of advanced glycation end products; ROS, Reactive oxygen species; SGLT-2, sodium glucose co-transporter-2; SOD, superoxide dismutase; TGF-β, transforming growth factor β; TNF-α, tumor necrosis factor α; TRAF6, TNF receptor associated factor 6; TSC2, tuberous sclerosis protein 2; ZO-1, Zonula occludens-1.
